# Public say food regulatory policies to improve health in Western Australia are important: population survey results

**DOI:** 10.1111/1753-6405.12128

**Published:** 2013-10

**Authors:** Christina M Pollard, Alison Daly, Michael Moore, Colin W Binns

**Affiliations:** School of Public Health, Curtin University, Western Australia; Department of HealthWestern Australia; School of Public Health, Curtin UniversityWestern Australia; Public Health Association of AustraliaACT; School of Public Health, Curtin UniversityWestern Australia

**Keywords:** food regulation, food policy, community attitudes, nutrition labelling, obesity

## Abstract

**Objective:**

To investigate the level of support among Western Australian adults for food control policies to improve diet, reduce obesity and protect the environment.

**Methods:**

Attitudes towards government food control policies on food labelling, food advertising, and the supply of environmentally friendly food data were pooled from two Nutrition Monitoring Survey Series telephone surveys of 2,147 adults aged 18–64 years collected in 2009 and 2012. Descriptive and logistic regression analyses were conducted using survey module of STATA 12.

**Results:**

The majority of adults believe it is important that government regulates food policy options under consideration: nutrition information on food labels (97% versus 2% who think it is not important); health rating on food labels (95% versus 3%); food advertising (83% versus 11%); and the supply of environmentally friendly food (86% versus 9%).

**Conclusions:**

Community perception is that government control or regulation of food labelling, food advertising and the supply of environmentally friendly food is important.

**Implications:**

Curbing excess weight gain and related disease burden is a public health priority. Australian governments are considering food regulatory interventions to assist the public to improve their dietary intake. These findings should provide reassurance to government officials considering these regulatory measures.

Recent food policy development has focused on all aspects of the food chain from production to consumption and has considered the economic, societal and environmental impacts of food.^1^ Poor diet, overweight and obesity, and subsequent chronic diseases such as heart disease, diabetes and some cancers are of increasing public health concern.^2^ Governments around the world are working to identify effective policy options to address food-related issues, and health is increasingly seen as an outcome of food policy, not just a driver.^3^ Food security and an environmentally sustainable food supply are also key food policy drivers.

There is increasing recognition that regulation has an important role to play as society addresses the public health problems of obesity and poor diet.^4^ However, this is in the context of increasing pressure on governments to use less regulation, as illustrated even by the name of the Australian Government's Department of Finance and Deregulation.^5^ The National Health and Medical Research Council's Australian Dietary Guidelines (Dietary Guidelines) assess the evidence to make dietary recommendations to protect against chronic disease. The Dietary Guidelines provide the policy context for nutrition-related regulatory assessment and form the basis for public nutrition education. The recent review of the guidelines highlight the need to achieve and maintain a healthy body weight by being physically active and choosing the appropriate amounts of nutritious food to avoid excess energy intake.^6^ The Dietary Guidelines also advise limiting intake of foods containing saturated fats, added salt and added sugars and, for the first time, they give specific recommendations to read food labels to identify healthier products.^6^ There is also evidence regarding the benefits of an environmentally sustainable food supply in terms of health, social and economic benefits.^7^ There is increasing evidence that dietary patterns consistent with the Guidelines provide health benefits and reduce the environmental impact associated with food. The food system uses land, water and energy. Outputs may include greenhouse gases, waste water, land deterioration, packaging and food waste. Many of these have significant environmental consequences for the food system.^6^

Obesity prevention strategies in Australia focus on improving diet and physical activity and consider, among other options, regulatory measures to improve diet (curbing inappropriate advertising and promotion of energy-dense, nutrient-poor foods and beverages on television and enhancing food labelling to support healthier food choices).^8^–^10^ The need to work with industry to ensure a more environmentally sustainable food chain is also identified.

Public health advocacy secures public and political support for legislation, policies and programs to promote health.11 Public opinion regarding policy options is part of the evidence needed to influence action as strongly supported options may be considered favourably by decision makers. In 2009, the Council of Australian Governments commissioned a review into front-of-pack nutrition labelling to assist consumers to make healthier food choices.^12^,^13^ In December 2011, Ministers from all jurisdictions and New Zealand, meeting as the Forum on Food Regulation, responded to Labelling Logic: Review of Food Labelling Law and Policy (2011) and requested “an interpretive front-of-pack labelling system be developed by December 2012 through a coordinated approach”.^14^ A project committee chaired by the Secretary of the Department of Health and Ageing was established to develop such a system.

The Department of Health in Western Australia conducts a Nutrition Monitoring Survey Series (NMSS) to assist with developing nutrition interventions to promote health, and monitors emerging food and nutrition policy issues for the Western Australian Government. This paper demonstrates the use of the NMSS to investigate the level of community support for the importance of government control or regulation over food policy options to improve diet and to reduce overweight and obesity among the Western Australian population. Support for government control or regulation over the supply of environmentally friendly foods is monitored, as this is an emerging consumer-driven and public health policy driver.

## Methods

### Sample

The Western Australian Nutrition Monitoring Survey Series (NMSS) uses computer-assisted telephone interviews to regularly collect information about nutrition-related attitudes and beliefs from randomly selected adults aged 18 to 64 years. The NMSS is conducted about every three years (1995, 1998, 2001, 2004, 2009 and 2012). This study uses the information collected in the two most recent surveys conducted in 2009 and 2012, as these were the only years the questions have been asked. For 2009 the sample size was 1,284 and for 2012 the sample size was 1,548. People listed in the most recent version of the Electronic White Pages (EWP) who met the age criteria of 18 and 65 were eligible to participate. A stratified random sample was extracted and all numbers called out. Mobile numbers are included in the EWP but anyone not listed in the EWP would not be included in the sample. In households with an adult in the target age range, the adult with the most recent birthday was selected to participate, and no substitutes were accepted. All sample numbers were called and accounted for in the response rates. Only respondents within the age range were included in the denominator and the average response rate over the two surveys was 83%. Both surveys were granted approval from the Western Australia Department of Human Research Ethics Committee.

The data were pooled and weighted for sample design and probability of selection in 2009 and 2012 and post survey adjustments were made to compensate for under or over representation of gender, age groups or areas of residence using the Estimated Resident Population for WA aged 18–64 years.

Any response bias associated with people who have telephones is addressed by the post estimation weighting, which adjusts to the total population so that estimates are about everyone including those without telephones. This is a common method used in surveys for this purpose.^15^ In addition, previous work on the differences in methods of survey administration found that the estimates from different methods were similar indicating that telephone surveys per se did not have a particular bias that was not shared by other methods where a telephone was not necessary.^16^

Table 1 describes the characteristics of the sample.

**Table 1:** Unweighted and weighted sample characteristics, NMSS 2009 and 2012 Western Australian adults

	Unweighted n	%	Weighted n	%
**Persons**	**2,832**	**100**	**2,832**	**100**

Female	1,835	64.8	1393	49.2

Male	997	35.2	1439	50.8

**Age grouped into three groups**				

18–30 years	461	16.3	1070	37.8

31–50 years	1,198	42.3	1003	35.4

51–64 years	1,173	41.4	759	26.8

Mean age in years	46	40		

**Area of residence**				

Metropolitan WA	1,976	69.8	2239	79.1

Rural WA	111	3.9	86	3.0

Remote WA*	745	26.3	507	17.9

Notes:

* Areas many kilometres away from large centres, mainly in the north west of the state

### Measures

The NMSS monitors population attitudes, knowledge and self-reported behaviours relating to food and nutrition policy, particularly the Australian Dietary Guidelines. The questionnaire contains a range of demographic and socio-cultural questions, as well as questions to measure community support for identified food policy options. Four questions were asked about government control over or regulation of food labelling, food advertising and environmentally friendly food supply: “How important would you say it is that the government has control over or regulates: (a) the supply of environmentally friendly food; (b) nutrition information on food labels; (c) health rating on food labels; and (d) food advertising. For each question, each of the five response options were read out to respondents: very important, quite important, neither important or nor important, quite unimportant, or not at all important. An additional open-ended question then asked for ‘other’ options. The ‘other’ was recoded back to the existing response categories where possible. No new themes emerged.

The socio-demographic variables collected included: age, gender, highest level of education attained, living arrangements, area of residence, annual household income, perceived discretional income, country of birth, employment status and SEIFA, which is a geographic area based index that reflects socio-economic advantage and disadvantage.^17^ Respondents were asked about a number of personal factors including their body weight and height and how concerned they were about the health aspects of their diet, and action they are taking regarding their weight and their fat intake. Perception about responsibility for food preparation, shopping, discretional income and their self-reported body weight and height were also collected.

### Statistical analysis

Analyses were conducted using the STATA 12.0 survey statistics module that adjusts for sample design and post estimation weighting. Pearson chi square tests were used to determine statistically significant differences for nominal data. Ordinal regressions were conducted for each of the five food control/regulation options. The full five-point Likert scale used to collect the data was used in the ordinal regressions. The direction of the ratings in the regression went from ‘not at all important’ (1) to ‘very important’ (5), with ‘very important’ used as the reference category.

Full models included all the variables as listed in Tables 2 to 4 and the inclusion of country of birth grouped into Australian born, UK/Ireland born and born in other countries; and employment status grouped as employed, unemployed, doing home duties, student, retired and other. The models were built in three stages. The first stage entered the socio-demographic variables (Table 2 plus country of birth and employment status) and retained all those with a *p* value of ≤0.1; then the attitudes were entered (attitudes towards health aspect of diet, fat and weight) and those with a *p* value of ≤0.1 were retained. The final stage entered the perception variables, who has responsibility for buying and preparing food, discretional income for each pay and BMI category. Interaction terms were included in the five models for household income and perceived discretional income. For the final models, all variables having a *p* value <0.05 were retained and reported. Post estimation for testing the validity of the proportional odds assumption was conducted using the STATA test Gologit2, with the svy option. Results from the five ordinal logistic regressions are expressed as Odds Ratios (OR) with 95% Confidence Intervals and *p* values.

**Table 2:** People who rated government food control or regulation (nutrition information on labels, health rating, food advertising, environmentally friendly supply) as quite or very important by socio demographic characteristics, NMSS 2009 & 2012 of Western Australian adults

	Nutrition information on food labels		A health rating on labels		Food advertising		Supply of environmentally friendly food	
				
	%	95% CI	%	95% CI	%	95% CI	%	95% CI
**Year of Survey**								
2009	97.5	96.2–98.4	92.8	90.4–94.6	85.3	82.6–87.7	84.6	[81.9,87.0
2012	96.7	95.4–97.6	94.9	93.0–96.3	83	80.5–85.2	85.4	[82.2,88.1

**Gender**								
Female	97.6	96.5–98.3	93.3	91.0–95.1	86.4	84.2–88.2	88	[85.8,89.9
Male	96.6	95.1–97.7	93.7	91.1–95.5	81.9*	78.9–84.6	81.9***	[78.3,84.9

**Age group**								
18-34 years	97.4	95.3–98.5	91.6	87.6–94.4	84.1	80.1–87.4	85.7	[81.3,89.2
31-50 years	97.4	96.1–98.3	95.2	93.2–96.7	84.9	82.3–87.1	85.9	[82.9,88.4
51-64 years	96.3	94.9–97.2	94	91.8–95.6	83	80.4–85.4	82.6	[79.2,85.5

**Highest education attained**								
Less than Year 12	96.2	93.9–97.7	96.1	93.7–97.7	86.4	82.0–90.0	85.7	[80.2,89.9
Year 12	97.7	95.3–98.9	93.3	87.5–96.5	84.1	78.6–88.4	83	[76.4,88.1
TAFE/Trade/Diploma	96.7	94.9–97.9	93.1	90.2–95.1	83.4	80.6–85.9	87.1	[84.0,89.6
Tertiary	97.5	96.0–98.4	93.3	90.2–95.4	84.3	80.8–87.2	83	[79.2,86.3

**Annual household income**								
Up to $60,000	96	92.7–97.9	93.4	89.3–96.0	96	92.7–97.9	86.9	[82.3,90.5
$60,001-$140,000	97.7	96.6–98.5	93.6	91.3–95.4	97.7	96.6–98.5	86.6	[83.7,89.0
Over $140,000	97.5	94.8–98.8	96	92.9–97.7	97.5	94.8–98.8	77.0*	[70.1,82.6

**Living arrangements**								
Living with children	97.3	96.0–98.1	94.6	92.8–96.0	84.7	82.4–86.8	86.5	[83.8,88.7
Living with other family/adults	97	95.5–98.0	92.6	89.4–94.9	83.8	80.7–86.5	83.6	[80.1,86.7
Living alone	96.5	93.8–98.0	90.2	83.5–94.3	80.1	73.5–85.3	80.4	[72.6,86.4

**Area of residence**								
Metropolitan Perth	97.3	96.3–98.0	94.1	92.1–95.6	85.4	83.3–87.3	85.9	[83.4,88.0
Rural areas of WA	98.8	94.7–99.7	90.5	79.5–95.9	75.9	67.6–82.6	91.6	[82.6,96.1
Remote areas of WA	96.1	93.5–97.7	91.5	88.2–94.0	79.7	75.1–83.7	79.5**	[75.1,83.4

**SEIFA Quintiles**								
Quintile 1	95.4	89.7–98.0	94.9	89.9–97.5	79	71.7–84.8	88.7	[82.6,92.8
Quintile 2	98.9	97.7–99.5	92.2	87.5–95.2	83.3	78.2–87.5	79.6	[73.0,84.9
Quintile 3	96.7	94.5–98.1	96.3	93.2–98.0	83.1	78.2–87.0	87	[82.5,90.5
Quintile 4	96.5	94.4–97.8	92.7	89.4–95.1	85.1	82.0–87.8	84.8	[81.1,87.9
Quintile 5	97.5	96.0–98.5	92.9	88.9–95.6	85.6	82.1–88.5	85.5	[81.2,89.0

Notes:

The p values (*p<0.05 **p<0.01 ***p0<.001) shown on the table are placed to show where there were statistically signficant differences in the univariate analysis between males and females; income and areas of residence in the rating of the importance of government control of food policy options.

**Table 4:** Rating government control or regulation as quite or very important for a health rating, nutrition information on labels, advertising, and environmentally friendly supply by perceptions related to food shopping, preparation, cooking Body Mass Index and discretional income, NMSS 2009 & 2012 of Western Australian adults

	A health rating on labels		Nutrition information on food labels		Food advertising		Supply of environmentally friendly food	
				
	%	95% CI	%	95% CI	%	95% CI	%	95% CI
**Responsibility for food shopping**								

No	93.9	86.0–97.5	95.9	92.4–97.8	80.3	73.8–85.5	85.5	78.6–90.5

Shared	93.2	91.0–94.9	97.7	96.7–98.4	84.8	82.5–86.9	86.3	83.6–88.6

Sole	93.6	91.1–95.5	97.0	95.6–98.0	84.8	82.1–87.2	83.6	80.3–86.5

**Responsibility for choosing and preparing meals**								

No	92.0	81.9–96.7	94.5	90.0–97.0	79.1	71.8–85.0	86.0	78.2–91.3

Shared	93.9	91.9–95.4	97.6	96.7–98.3	84.3	81.9–86.4	86.3	83.5–88.6

Sole	93.6	91.3–95.4	97.4	96.0–98.3	85.2	82.5–87.5	83.8	80.6–86.5

**Cooking skills**								

None or more basic meals	93.1	88.9–95.8	96.6	94.6–97.9	81.6	77.3–85.2	83.4	78.7–87.2

A wide variety of meals	93.9	91.2–95.8	98.5	97.5–99.1	86.8	84.2–89.1	86.2	83.0–88.9

Almost anything	93.3	90.7–95.2	95.5*	93.3–97.1	82.4*	79.2–85.2	84.4	80.8–87.4

**Discretional income from each pay**								

Don't earn enough to save	93.9	89.4–96.6	97.1	95.3–98.2	87.1	83.8–89.8	89.0	85.1–92.0

Earn enough to save a bit	93.6	91.5–95.2	97.4	96.3–98.3	83.2	80.7–85.4	85.1	82.5–87.3

Earn enough to save a lot	92.9	87.7–96.0	96.0	92.5–97.9	83.9	78.9–87.8	77.3***	68.8–84.0

**BMI Category**								

Not overweight/obese	93.0	89.3–95.5	98.7	97.5–99.4	86.0	82.5–88.9	88.0	84.3–90.9

Overweight	94.1	91.5–95.9	96.4	94.6–97.6	82.2	79.2–84.8	82.0	78.3–85.1

Obese	93.0	90.0–95.1	96.3***	94.5–97.6	85.0**	81.9–87.7	85.0	81.0–88.3

Notes:

The p values (*p<0.05; **p<0.01; ***p<0.001) shown on the table are placed to show the statistically significant differences in the univariate analysis between the categories of cooking skills and BMI and rating of government control of nutrition information and advertising and the perceived discretional income categories and rating the government control of food policy.

## Results

Tables 2, 3 and 4 present the univariate descriptive results in terms of proportion of the people surveyed who ranked government control or regulation of each of the food policy areas as either quite or very important by socio-demographic variables (Table 2); attitudes towards diet and action being taken regarding their weight and fat intake (Table 3); and perception about food buying and preparation, amount of discretional income from each pay period and Body Mass Index category based on self-reported height and weight and corrected for over-reporting of height and under-reporting of weight^11^ (Table 4). The results for the policy areas are presented below.

**Table 3:** People who rated government food control or regulation (nutrition information on labels, health rating, food advertising, environmentally friendly supply) as quite or very important by selected opinions, NMSS 2009 & 2012 of Western Australian adults

	Nutrition information on food labels		A health rating on labels		Food advertising		Supply of environmentally friendly food	
				
	%	95% CI	%	95% CI	%	95% CI	%	95% CI
**Best describe your attitude to the health aspects of your diet**								

Pay lot of attention	93.6	90.9–95.5	97.8	96.5–98.6	88.1	85.8–90.1	86.0	82.9–88.5

Take a bit of notice	94.5	92.6–96.0	97.3	96.0–98.2	83.1	80.4–85.5	84.9	81.9–87.4

Don't think about it	86.1	73.4–93.3	92.0**	85.5–95.7	68.3***	57.7–77.2	78.5	66.7–86.9

**What you are doing about your fat intake**								

Trying to eat less	95.2	92.5–96.9	97.7	96.1–98.6	85.0	81.7–87.8	85.0	81.0 –88.2

Thinking about eating less	92.5	87.1–95.7	96.6	93.6–98.3	80.2	75.4–84.3	87.2	82.6–90.8

Not thinking of eating less	87.2	78.6–92.6	96.0	91.3–98.2	79.5	72.1–85.3	75.9	67.2–83.0

I already eat a low fat diet	94.4	92.2–96.0	97.1	95.9–98.0	86.4	83.8–88.7	86.4	83.4,89.0

**What you are doing about your weight**								

Trying to change	95.1	92.9–96.6	96.2	94.4–97.4	83.6	80.7–86.1	83.8	80.4–86.8

Thinking of trying change	92.7	86.7–96.1	97.4	95.3–98.6	84.9	80.6–88.4	86.2	81.6–89.8

Not thinking of change	92.3	89.4–94.4	97.8	96.7–98.6	84.3	81.3–86.9	85.4	82.2–88.2

Notes:

The p values (**p<0.01 ***p<0.001) shown on the table are placed to show the statistically significant differences in the univariate analysis between attitudes toward diet and the rating of importance of government control of food policy options.

### Regulation of food labelling

The results show that across the population, no matter which way the data is examined, government control or regulation of nutrition information and a health rating on food labels is considered important. Overwhelmingly, people want government control over the nutrition information on the label, with 97.1% of the population surveyed reporting that government control or regulation of nutrition information on food labels is quite (21.0%) or very (76.1%) important; and 92.8% reporting that the government control or regulation of a health rating on food labels is quite (24.4%) or very (69.2%) important. Table 2 shows that there is no one socio-demographic group, attitude or perception that is statistically significantly associated with government controlling or regulating what is on nutrition information or health ratings on labels. Additional suggestions about what should be controlled on food labels were made by 2.3% of those surveyed. These comments were about the inclusion and control of information concerning additives, e.g. “Food additives should be much more tightly controlled and there are too many loopholes for additives to not be declared on labels”, or about the source of food, e.g. “Regulate products to have writing on the label saying where the food is grown not only where it is produced”.

Results from the two ordinal regression models conducted for the rating of importance for controlling or regulating nutrition information on food labels show that, when all variables were accounted for, the rating decreased if people were not living in metropolitan area (OR 0.76 [0.67–0.86] *p*<0.0001) and if they paid less than “a lot of attention to the health aspect of their diet” (OR 0.68 [0.56–0.83] *p*<0.0001). People surveyed in 2012 were also 14% less likely to rate controlling nutrition information labelling important compared with 2009 (OR 1.14 [1.05–1.23] *p*<0.001).

### Regulation of food advertising

Eighty-four per cent people surveyed reported that government control or regulation of food advertising was either quite important (34.0%) or very important (50.1%). Specific comments about the control of advertising in relation to fast food, e.g. “Advertising of high fat, take away foods”, particularly with regard to children e.g. “Advertising aimed towards children in regard to junk, fast food”; “Advertising in children's time slots”; and “Advertising to children, like ads that target children” were added by about 1% of those surveyed. The socio-demographic Table 2 shows that men and people living in rural areas of WA are most likely to give lower importance ratings to food advertising and Tables 3 and 4 show that people who do not pay a lot of attention to the health aspects of their diet, who have a BMI above a healthy weight range and who report very good cooking skills are also more likely to give lower importance ratings to food advertising.

Results from the ordinal regression showed that people who reported that they could “save a lot of money” each pay were associated with decreased likelihood of rating the importance of government control or regulation of food advertising highly (OR 0.81 [0.69–0.95] *p*<0.001). People living in rural areas of WA were 44% less likely to give high ratings of importance to government control or regulation of food advertising and people living in remote areas of WA were 21% less likely (OR 0.56 [0.37–0.84 *p*<0.01] and OR 0.79 [0.63–0.99 *p*<0.05] respectively). Attention to diet was a major association with decreased likelihood of rating importance of regulating food advertising highly. People who reported that they only paid a bit of attention to the health aspect of their diet were 29% less likely to rate the importance of regulating food advertising highly (OR 0.71 [0.58–0.86] *p*<0.0001) and people who paid no attention to the health aspect of their diet that were 59% less likely to rate government control or regulation of food advertising highly (OR 0.41 [0.25–0.66] *p*<0.0001).

### Environmentally friendly food supply

Eighty-five per cent of people surveyed reported that government control or regulation of an environmentally friendly food supply was either quite important (38.3%) or very important (46.6%). The socio-demographic Table 2 shows that males, households with an income over $140,000 and people living in rural areas of WA are most likely to give lower importance ratings to the supply of environmentally friendly food. Tables 3 and 4 show that people who have the ability to “save money from each pay” are also more likely to rate the supply of environmentally friendly food as less important.

Results from the ordinal regression for the importance of government control or regulation of the supply of environmentally friendly found three statistically significant associations. Men were 31% less likely to rate government control or regulation of an environmentally food supply compared with women (OR 0.69 [0.51–0.92] *p*<0.05) and there was decreasing likelihood of rating importance high with the reported ability to save a little (OR 0.67 [0.47–0.71] *p*<0.05) or a lot (OR 0.40 0.23–0.71] *p*<0.01). Being born in a country outside Australia was associated with a 38% increased likelihood of a higher rating of importance compared with those born in Australia (OR 1.38 [1.01–1.90] *p*<0.05).

## Discussion

The present study is unique in its assessment of public perceptions of the importance of government control or regulation over policy options currently under consideration. The study design, which includes collection of socio-demographic characteristics as well as self-reported behaviour, attitudes and perceptions, enables assessment of the possible influence of these factors across the adult population of Western Australia.

Across Western Australia and within socio-demographic groups of differing attitudes and perceptions, the perceived importance of government control or regulation of food labelling, food advertising, and supply of environmentally friendly food is high and widespread. The results from the ordinal regression show minimal statistically significant associations with how people rate the importance of government control over the policy areas, which provides further evidence for the general community support for these interventions.

### Nutrition information and health rating on food labels

Nutrition information on food labels is considered a credible and prominent source of nutrition information by consumers.^19^ It is not surprising that consumers are interested in government control of this information. Our current study findings demonstrate that consumers think it is important that government controls or regulates nutrition information directed at the public.

The findings support the recommendations of the Australian Government's independent review of food labelling that examined policy drivers, considered government's role, and identified principles and approaches for appropriate enforcement.^12^ The review reinforced the principle that public health and safety is the main policy driver for food labelling and that there is community support for high levels of regulation for the management of public health issues. Consumers demanded accurate, consistent and adequate information on food safety, healthy eating, technical issues (e.g. genetically modified foods), and ethical issues (e.g. country-of-origin); while industry policy drivers related to marketing needs, trade facilitation and industry viability.^12^ Although Australia has had mandatory nutrition information panels on all food products since 2002, to support public health, the review recommended the inclusion of explicit nutrition information on labels with simplified presentation to further assist healthy food choices.^12^

Previous Australian research asking about support for food-related policy issues found that 84% of respondents paid attention to food labels, and 87% supported a traffic light labelling on food packaging. Importantly, 88% said they would use this information when making food selections.^20^

It is instructive to compare successful Australian tobacco product labelling, which has used a combination of legislative, policy and program interventions, with the current status of nutrition labelling.^11^ Despite strong evidence about the negative health impacts of smoking being available since the 1950s, government was slow to act. However, in 2012, Australia introduced plain packaging of tobacco products, five years after requiring that 30% of the front of a tobacco pack and 90% of the back of the pack carry extensive mandatory health warnings.^21^ Public support for regulatory interventions relating to tobacco control was often ahead of current government practices.^22^ The success of using labelling of products as part of the mechanisms that have been successful in dealing with the health impacts of tobacco suggests that with similar community support this sort of mechanism may be able to be adapted as part of an approach to dietary-related illness. Our findings indicate that with the high perceived importance related to food labelling, there would be even stronger community support for each of the food regulatory options that are presented above. On 14 June 2013, the Australian and New Zealand Food Regulation Ministers announced that a voluntary ‘Health Star Rating’ front of pack labelling system would be implemented.^23^ There is a welcomed caveat for its implementation: if, after two years of implementation, the system is not consistent, widespread and effective, a mandatory approach would be required.^23^

### Food advertising

Mechanisms exist at state and national levels to control some aspects of food advertising.^24^ In Australia there are a number of government regulatory controls and industry voluntary controls on food advertising, particularly relating to misleading content.^25^,^26^ Increasingly, governments are considering regulatory options to reduce food advertising.^27^ Morely et al. found that 83% of Australians surveyed favoured a ban on the advertising of unhealthy food to children during their viewing times, however, only 56% supported a total ban on unhealthy food advertising at all times.^20^ As has been found in previous studies, there was higher support for interventions that were perceived to protect children. The authors cautioned about low response rates in the previous study and possible desirability bias, however, our current findings reinforce their findings.^20^ There appear to be some regulatory options to curb television advertising at state level and a number of jurisdictions have considered taking action. Considering the high levels of support for regulation and the levels of concern by governments over poor diet, overweight and obesity, and subsequent chronic diseases such as heart disease, diabetes and some cancers, these findings should provide a catalyst to support early government action.

### Supply of environmentally friendly food

There are high levels of community support for the importance of government having control over supply of environmentally friendly food. Food policy makers are urged to consider options that improve the health of the population and are favourable to the environment and sustainable development.^28^ Strategies to ensure a sustainable food supply include integrating food policy actions to address its determinants, such as protecting the environment by monitoring the environmental impact of food production or decreasing food wastage.^6^ Actions can be taken at an individual as well as at an industry or government level.^3^,^29^

Although health is one of the outcomes of an environmentally friendly food supply, the responsibility for government control or regulation over environmental measures lay with a number of sectors outside health (including agriculture, planning, transport, trade, and commerce), highlighting the need for intersectoral collaboration across government, as in the 1992 Food and Nutrition Policy^30^ and the recent National Food Plan.^31^ However, this plan provides encouragement and incentives, for example, to reduce food waste through the National Waste Policy, (which includes a food and garden organics best practice collection manual, supermarket food waste benchmarks and national food waste assessments), without considering regulation. In lay terms, this translates to a supply of environmentally friendly food. How to achieve this is complex, but the results of this present study indicate that the community thinks this is an important food policy for government control and regulation measures.

Maintaining public trust is an important issue for governments developing food and nutrition policy. Previous research has identified that the gap between production and consumption has led to a decline in consumer trust in food and a desire for increased regulation.^32^ This is at odds with the desire for deregulation among some members of the food industry.

The findings of this research show congruence between the current food control or regulatory options being considered by government – such as nutrition information and education on food packaging, front of pack labelling (health rating on foods) and restricting television advertising of foods high in fat, salt and added sugars – and consumer perceptions of the importance of government control of these options. Trust is a key element in food choice and is integral to public attitudes to food regulatory measures to improve public health.^33^

There are strengths and limitations to this survey. The high response rate and the representative sample selection mean that the results can generalised to Western Australia. Social desirability may have influenced some responses. There is considerable vested interest in some areas of the food industry in building marketing, promotion and sale of poor food choices. Any bias related to missing people without phones is not particularly related to the CATI method but rather to being surveyed per se as suggested by other research.^16^ However, this type of self-reported survey information is useful evidence to measure public opinion for advocacy campaigns and is essential for public health policy planning. While the survey did not ask directly about support for government regulation of food, the very consistent and high level of importance reported for such suggests that any control or regulation would be supported by a large majority of Western Australians. Our study findings are consistent with previous Australian research relating to some of these policy issues, suggesting the likelihood that similar views would be held across Australia.^20^

## Conclusion

These findings suggest there would be widespread support for government actions to regulate food labelling and promotion for the protection of public health. The public health and consumer information policy drivers support taking regulatory action for nutrition information and a health rating on food labels. There is also likely to be widespread community support for taking action for the supply of environmentally sustainable or friendly food.
